# Valvar replacement in infants and preschool children: a retrospective single-center study

**DOI:** 10.1186/1749-8090-8-S1-P173

**Published:** 2013-09-11

**Authors:** G Liguori, A Kanas, J Penha, L Miana, L Caneo, C Tanamati, M Jatene

**Affiliations:** 1 Heart Institute (InCor), University of Sao Paulo Medical School, Sao Paulo, Brazil

## Background

The use of prosthetic valves in children is controversial, being indicated only if valve repair is not possible. In this study, we sought to report and evaluate the immediate and long-term outcomes of our experience in valvar replacement in infants and preschool children.

## Methods

We conducted a retrospective study of 16 infants and preschoolers patients who undergone valve replacement between 1997 and 2006. We analyzed medical records, echocardiographic exams and surgical reports.

## Results

Mean age at surgery was 11.7±10.4 (min=0.8, max=32.9) months. Diagnoses were isolated congenital valvopathy (38%), atrioventricular septal defect (AVSD) (31%), common arterial trunk (19%) and tetralogy of Fallot (12%). Nine (50%) of the prostheses were located in mitral, 4 (22%) in aortic, 2 (11%) in pulmonary and 3 (17%) in truncal position. Five (31.3%) patients underwent reoperation during the follow-up period and the major indication was prosthetic valve stenosis. The mean time free of reoperation was 76.5±18.8 months. Hospital mortality was 43.8% and the average survival time 58.4±9.3 months. Statistically significant association was found between age at surgery and the occurrence of intra-hospital death, the younger the patient, the greater the risk of dying during the first month after the surgery (p=0.026). There were differences between the intra-hospital mortality risks according to the underlying congenital heart disease, although this difference was not statistically significant (p=0.084). Valvopathies due to other cardiac defects (AVSD, common arterial trunk and tetralogy of Fallot) had higher occurrence of intra-hospital death.

## Conclusion

Valve replacement in infants and children in preschool age, although should be reserved for cases in which there is no possibility of valvuloplasty, proved to be an immediate therapeutic alternative. Still, reoperations are needed and the duration of the valve prosthesis quite variable.

